# Ultrasound-Assisted Fibril Formation Enhances Complexation of Oat Globulin with Quercetin: Mechanism, Structure Evolution, Delivery Performance

**DOI:** 10.3390/foods14223916

**Published:** 2025-11-16

**Authors:** Jinzhao Xu, Xiao Zhao, Qingfeng Ban

**Affiliations:** 1 Key Laboratory of Dairy Science, College of Food Science, Ministry of Education, Northeast Agricultural University, Harbin 150030, China; 2 College of Equipment Management and Support, Engineering University of People’s Armed Police, Xi’an 710086, China

**Keywords:** plant protein, amyloid fibrillation, ultrasound, polyphenol delivery, bioaccessibility

## Abstract

Amyloid fibrillization represents an effective strategy for extending and enhancing protein function, particularly for the delivery of hydrophobic active substances. In this study, oat globulin (OG) and its fibrils were complexed with quercetin (Que) to construct the delivery system, and ultrasonic pretreatment was applied during fibril preparation to explore the promoter of complex formation. The results demonstrated that complexation with Que induced a dose-dependent static quenching of the intrinsic fluorescence of the protein/fibrils, with hydrophobic interactions and tryptophan residues being the primary interaction forces and the main fluorescence quenching groups, respectively. In comparison, OG fibrils prepared with ultrasound pretreatment (UOGF) exhibited the strongest encapsulation and loading capacity for Que, ranging from 97.16% at a mass ratio of 200:1 to 42.48% at a ratio of 25:1. Subsequently, complexes were prepared with a ratio of 50:1. Structural analysis revealed that Que primarily interacts with the protein/fibril carriers through hydrogen bonds and hydrophobic interactions, inducing structural changes and ultimately being encapsulated in an amorphous form within the composite material. Additionally, Que promoted the mutual aggregation and cross-linking of protein/fibril units, leading to increased hydrodynamic diameter and zeta-potential. Moreover, UOGF-Que showed the greatest improvement in the thermal stability and the photostability of Que, and enhancing the bioaccessibility. These findings provide valuable insights into using ultrasound as an auxiliary measure for fibril self-assembly to enhance the application potential of fibrils, especially the delivery of hydrophobic functional substances.

## 1. Introduction

Bioactive natural compounds are valuable resources for developing functional foods. Quercetin (3,4,5,7-tetrahydroxyflavonol, Que) is a flavonoid polyphenol compound that features both hydrophilic hydroxyl groups and a hydrophobic ring structure [1,2]. Que is abundant in various natural plants, including onions, apples, berries, and green tea, as well as in medicinal plants such as *Hypericum perforatum* and *Ginkgo biloba* [3,4]. Que possesses several health-regulating functions, including immune regulation [5], anti-allergic effects [6], cardiovascular protection [7], prevention of neurological diseases [8], anti-angiogenic and anti-cancer activities [9,10]. Additionally, Que has antioxidant properties, effectively scavenging free radicals, well-known antibacterial and antiviral activities [11,12]. However, due to Que’s low water solubility, poor bioavailability, and chemical instability in physiological conditions, its use as a health promoter in the food and pharmaceutical industry is still limited [13,14]. Previous reports indicate that Que’s solubility in water is approximately 0.01 mg/mL at 25 °C [15]. Furthermore, Que may oxidize or degrade when exposed to UV light, high temperatures or extreme pH conditions [16]. To overcome these limitations, delivery systems based on biomolecules have been developed to improve the Que’s bioavailability, such as emulsions [17], liposomes [18], hydrogels [19], microcapsules [20], and polymer nanoparticles [21].

Food proteins are widely used as delivery substrates for bioactive substances due to their nutritional functions, degradability, and biocompatibility [22]. Amyloid fibrils are a unique protein structure and are regarded as a promising approach to enhance and extend protein capabilities [23,24]. Compared to native proteins or random aggregates, fibrils exhibit structural features that endow them with extremely high aspect ratios, superior structural stability, collective ordering properties, and high specific surface areas, along with numerous biological functions [25]. The process of fibril formation is also accompanied by the reorganization of active sites and hydrophobic regions within the molecular structure, thereby facilitating interactions with bioactive substances and enhancing functionality [26,27]. Additionally, fibrillar supramolecular structures demonstrate stability and compactness in acidic environments, whereas alkaline conditions may induce controlled structural fragmentation or dissociation [27]. Therefore, protein fibrils are promising candidates for delivery systems.

The properties of the delivery system are often influenced by the structural phenotype and species of the protein carrier. Protein type affects the fibril assembly behavior and the availability of interaction sites [28]. In previous, fibrils from soybean, peanut, pea, mung bean, and potato proteins showed significantly different interaction strengths and complexing behavior when binding to astaxanthin, leading to differences in delivery efficiency [29]. Oat globulins (OGs) have shown promise as a resource for fibril production and have potential for active substance delivery [30]. OG is a sustainable plant protein resource, featuring outstanding advantages of easy accessibility, high nutritional value, and low allergenicity [31]. Fibrillation further enhances their application potential, such as cross-linking network construction, antioxidant capacity, interfaces, modifiable properties, etc. [30,32]. Our previous study showed ultrasound pretreatment before fibrillation process effectively promoted the acid hydrolysis and primary nucleation of OG proteins in the subsequent fibrillation process under acid–heat conditions [25]. Ultrasonic pretreatment also indirectly affects the aggregation behavior and structural assembly mode of OG fibrils, as well as changes in the composition of the core building peptides. The differentiated fibril structures caused by the differences in self-assembly construction behavior during fibrillation, such as elongated or worm-like profiles, have a considerable impact on the loading capacity and sustained release effect of the target substance [33]. Therefore, it is indispensable to clarify the relationship between the structural modification of protein fibril carriers by ultrasound technology and the resulting interaction with functional substances.

Based on these considerations, this study aimed to construct gastrointestinal delivery systems by complexing OG and its fibril derivatives with Que. Assuming that specific structural alterations of fibrils influence their encapsulation and binding capacities with targets, ultrasound-assisted fibrillation was employed to enhance carrier performance. Three protein/fibril forms were selected as the primary objects: OG, conventionally prepared fibrils (OGF), and ultrasound-pretreated fibrils (UOGF). Initially, multiple spectroscopic techniques were employed to elucidate molecular interactions, binding capabilities, and thermodynamic properties of three protein/fibril forms during the complexation process with Que. Subsequently, the effects of the three carrier systems on Que encapsulation were investigated, along with structural and micromorphological changes throughout the complexation process. This may provide insights into promoting the application of protein fibrils in efficient delivery systems for bioactive compounds.

## 2. Materials and Methods

### 2.1. Materials

Oats were sourced from Heilongjiang Cang Natural Ecological Agriculture Co., Ltd. (Harbin, China). Quercetin (Que), Thioflavin T (ThT), pancreatin, and pepsin were obtained from Sigma-Aldrich Chemical Co. (St. Louis, MO, USA). Congo red (CR), Porcine bile extract, and other chemical reagents were purchased from Macklin (Shanghai, China). All chemicals used were of analytical grade.

### 2.2. Preparation of OG Fibrils

OG and OGF were prepared according the procedure described in a previous study [30]. Additionally, prior to fibrillation, the OG solution (30 mg/mL, pH 2) was subjected to pretreatment using an ultrasound generator (Scientz-II D, Scientz Biotechnology Co. Ltd., Ningbo, China) with a titanium alloy probe (6.0 mm diameter). For ultrasound pretreatment, samples were sonicated at the power density of 9 W/mL for 20 min with a frequency of 20 kHz and a pulsed mode (5 s on, 5 s off). This process was performed in a brine ice-water bath to maintain the temperature below 15 °C and effectively dissipate the heat generated during sonication. Subsequently, the ultrasonicated OG solution was heated in a thermostatic oil bath at 90 °C for 18 h to obtain ultrasound-assisted OG fibrils (UOGF).

### 2.3. Characteristics of OG Fibrils

#### 2.3.1. Analysis of Fluorescence Quenching Binding Mechanism

Intrinsic fluorescence spectroscopy was employed to investigate the interaction between OG and its fibrillar products with Que. The binding capacities of OG, OGF, and UOGF were evaluated at pH 3.2 with various concentrations of Que (0, 2.5, 5, 10, 15, 20, and 30 μM). The mixed samples were incubated at different temperatures of 24.85, 30.85, and 36.85 °C (298, 304, and 310 K) for 30 min. Then the intrinsic fluorescence spectra of each sample (0.3 mg/mL) were recorded using a Cary Eclipse fluorescence spectrophotometer (Agilent Technologies, Santa Clara, CA, USA) following to the previously described protocols [34,35]. The instrument voltage was set to 650 V, with a slit width and scan rate of 5 nm and 1000 nm/min, respectively. Meanwhile, to correct for the inner filter effect, the following equation was applied as described by Ren et al. [36].

(1) $$F_{c}=F_{m}\times 10^{{(A}_{ex}+A_{em})/2}$$

where *F_c_* and *F_m_* represent the corrected and measured fluorescence intensities, while *A_ex_* and *A_em_* denote the absorbance at the excitation and emission wavelengths, respectively.

The fluorescence quenching mechanism of the complex system was further analyzed by fitting the obtained fluorescence intensity data using the Stern–Volmer equation:

(2) $$\frac{F_{0}}{F}=1+K_{\mathrm{SV}}\left[Q\right]=1+K_{q}\tau_{0}\left[Q\right]$$

where *F*_0_ and *F* represent the fluorescence intensities of OG and its fibrils before and after the addition of the polyphenol quencher (Que), respectively; *K_SV_* is the Stern–Volmer quenching constant; *K_q_* is the quenching rate constant; [*Q*] is the concentration of the quencher Que; and *τ*_0_ is the average lifetime of the fluorophore in the absence of the quencher.

#### 2.3.2. Synchronous Fluorescence Spectroscopy

Synchronous fluorescence spectroscopy was used to characterize the effect of Que on the microenvironmental changes in Tyr and Trp residues in OG and fibrils. The Que concentrations were set at 0, 2.5, 5, 10, 20, 30, and 40 μM. The wavelength intervals (Δλ) were set at 15 nm and 60 nm for Tyr and Trp, respectively. The voltage, slit width, and scanning rate settings were consistent with those used for intrinsic fluorescence spectroscopy, and the detection temperature was set at 24.85 °C.

#### 2.3.3. Determination of Fibril Conversion Rate

The fibril conversion rate was determined following the procedures described by a previous report with slight modifications by Hu et al. [37] and Zong et al. [38] with slight modifications. Briefly, the fibril sample was diluted to a concentration of 5 mg/mL and transferred into an ultrafiltration centrifuge tube (100 kDa MWCO, Merck Millipore Ltd., Cork, Ireland). The sample was centrifuged at 4000× *g* for 15 min at 4 °C, and this process was repeated three times. The protein concentration of the filtrate was subsequently measured using the Bradford assay (P0006, Beyotime, Shanghai, China) following the instructions provided. The conversion rate of fibrils was calculated according to the following formula:

(3) $$Conversation\;rate\;\left(\%\right)=\frac{C-C_{F}}{C}$$

where *C* was the original concentration of OG protein and *C_F_* was the filtrate concentration.

#### 2.3.4. Determination of Hydrolysis

The degree of hydrolysis was determined using the o-phthaldialdehyde (OPA) method with slight modifications [39]. Briefly, the sample (200 μL) was mixed with 1.5 mL of OPA reagent and incubated in the dark for 2 min. Serine solution (0.952 meqv/L) was used as the standard. The absorbance was then measured at 340 nm, and the DH was calculated according to the following equations:

(4) $${{Ser}_{eqv}\;NH}_{2}=\frac{A_{sample}-A_{blank}}{A_{standard}-A_{blank}}\times \frac{\left(0.9516\times V\times 100\right)}{\left(X\times P\right)}$$

(5) $$DH\left(\%\right)=\frac{{Serine\;NH}_{2}-\beta }{\alpha \times h_{tot}}\times 100\%$$

where *Ser_eqv_ NH*_2_ is the serine equivalent of protein; *V* is the sample volume (L); *X* is the sample weight (g); *P* is the protein content (%); *α* and *β* are constants with values of 0.970 and 0.342, respectively; and *h_tot_* is the total number of peptide bonds in OG.

### 2.4. Preparation of Oat Protein/Fibril Complexes with Quercetin

Firstly, OG, OGF, and UOGF solutions were diluted to a protein concentration of 5 mg/mL and adjusted to pH 3.2 using 1 M NaOH solution to mimic acidic food and beverage conditions [40]. Under stirring conditions, ethanol-dissolved Que stock solution (10 mg/mL) was slowly added to the protein/fibril solution (5 mg/mL) and continuously stirred in the dark for 6 h. The final ethanol concentration in the mixture was maintained below 2% (*v*/*v*) to avoid affecting the protein structure [27]. The final Que concentrations in the complex system were 0.025, 0.05, 0.1, 0.15, and 0.2 mg/mL, corresponding to protein/fibril-to-Que mass ratios of 200:1, 100:1, 50:1, 33.3:1, and 25:1, respectively. Additionally, to further explore the structural properties and delivery capacity of the complex system, a final Que concentration of 0.1 mg/mL was added to the OG and fibril solutions (5 mg/mL) to achieve a mass ratio of 50:1. The resulting complex solutions were stored at 4 °C or lyophilized for further analysis. The OG, OGF, and UOGF complexes with Que were denoted as OG-Que, OGF-Que, and UOGF-Que, respectively.

### 2.5. Determination of Encapsulation Efficiency and Loading Capacity

The encapsulated Que in complex system were determined by monitoring the dissolved portion based on previous report with slight modifications [41]. The UV absorption spectrum of Que was recorded between 300 and 650 nm, with λ_max_ determined at 373 nm. High-speed centrifugation (8000× *g*) was used to separate the precipitated Que from the protein/fibril–Que complexes. The collected supernatant was mixed with anhydrous ethanol at a ratio of 35:65 (*v*/*v*) and subjected to ultrasonication for 5 min to fully extract the dissolved Que. The UV absorption value at 373 nm was measured using a UV-Vis spectrophotometer, and a Que standard curve was established using 65% ethanol aqueous solution. The encapsulation efficiency (%EE) and loading capacity (LC) of Que were calculated using the following formulas:

(6) $$EE\left(\%\right)=\frac{M_{supernatant}}{M_{total}}\times 100\%$$

(7) $$LC\left(\%\right)=\frac{M_{supernatant}}{Carrier\;mass}\times 100\%$$

where *M_supernatant_* represents the amount of encapsulated Que in the supernatant, *M_total_* is the total amount of added Que, and *Carrier mass* is the total amount of protein/fibril carriers.

### 2.6. Characterization of Cross-β Structural Changes in Fibrils

Congo red (CR) staining was used to characterize fibril-specific structural changes based on previous research with slight modifications [42]. CR (7 mg) was dissolved in 10 mL phosphate buffer (10 mM, pH 7.0, containing 150 mM NaCl) and filtered through a 0.22 μm membrane to obtain a CR stock solution. The CR stock solution was diluted 20-fold with phosphate buffer (10 mM, pH 7.0) to prepare a CR working solution (50 μM). For measurement, 40 μL of the sample was mixed with 4 mL CR working solution and incubated in the dark at 25 °C for 30 min. The absorbance spectrum was recorded in the wavelength range of 400–600 nm using a UV spectrophotometer.

Additionally, ThT fluorescence spectroscopy was performed following the previously described methods [43,44]. Briefly, the sample (47 μL) was mixed with 4 mL of the ThT working solution and incubated for 3 min. The fluorescence spectra were recorded in the range of 460–580 nm using a Cary Eclipse fluorescence spectrophotometer (Agilent Technologies, USA) with an excitation wavelength of 440 nm.

### 2.7. ATR-FTIR Spectroscopy

FTIR spectra of freeze-dried samples were recorded using a Nicolet-IS10 infrared spectrophotometer (Thermo Scientific, Waltham, MA, USA) equipped with an attenuated total reflection (ATR; diamond crystal) accessory. The sample was evenly stacked on the ATR module and compressed using a rotating press to ensure tight contact with the crystal. Spectra were collected in the range of 4000 cm^−1^ to 525 cm^−1^ with a resolution of 4 cm^−1^. The obtained spectra were processed through OMNIC 8.2 software (Thermo Scientific), including the following steps: correction of ATR, baseline correction, and normalization.

### 2.8. Size Distribution and Zeta-Potential Measurement

The samples were diluted to 0.3 mg/mL (pH 3.2) with Milli-Q water and 6 M HCl. The hydrodynamic diameter and Zeta-potential of the samples were analyzed using a Zetasizer Nano ZS (Malvern Instrument Ltd., Worcestershire, UK). All measurements were conducted at 25 °C and repeated in triplicate, with an equilibration time of 120 s.

### 2.9. Transmission Electron Microscopy (TEM)

The microstructural of OG, OGF, and UOGF before and after complexation with Que were examined. The sample solutions were diluted to 0.1 mg/mL (pH 3.2), deposited onto carbon-coated copper grids, and incubated for 10 min. The grids were then stained with 2% uranyl acetate. The morphology of the samples was observed using a HT7800 transmission electron microscope (Hitachi Inc., Tokyo, Japan) operating at an acceleration voltage of 100 kV.

### 2.10. Stability Analysis of Quercetin

The thermal stability of Que was evaluated following a previously described method with slight modifications [45]. Briefly, 5 mL aliquots of free Que suspension (control), OG-Que complex solution, OGF-Que complex solution, and UOGF-Que complex solution were placed in a water bath at 85 °C under dark conditions in sealed containers. At predetermined time intervals (0, 10, 30, 60, 90, 120, and 150 min), samples were withdrawn, rapidly cooled to 25 °C, vortexed, and analyzed for Que retention.

The photostability of encapsulated Que was assessed using a 365 nm UV lamp with a light intensity of 1.32 mW/cm^2^. Aliquots of 5 mL from each sample group were placed 10 cm away from the UV light source and exposed for various durations (0, 10, 30, 60, 90, 120, and 150 min). The retention of Que in each sample was then measured.

### 2.11. In Vitro Digestion Characteristics

The release behavior and bioaccessibility of Que in the complex system were evaluated using an in vitro simulated digestion model with modifications based on previous studies [45,46]. Briefly, sample solutions loaded with Que (protein concentration: 4 mg/mL) were mixed with equal volumes (10 mL) of simulated gastric fluid containing pepsin. The pH was adjusted to 2.0 using 6 M HCl, and the mixture was incubated at 37 °C with shaking at 100 rpm to simulate gastric digestion for 2 h. Afterward, the pH was adjusted to 7.0 to terminate gastric digestion. For intestinal digestion, 5 mL of the gastric digest was mixed with 5 mL of simulated intestinal fluid containing trypsin and bile salts, followed by incubation at 37 °C for another 2 h. The collected digestion products were centrifuged at 10,000× *g* for 30 min at 4 °C, and the supernatant was analyzed for Que content to determine the bioaccessibility:

(8) $$Bioaccessibility\;of\;Que\left(\%\right)=\frac{C_{supernatant}}{C_{digesta}}\times 100\%$$

where *C_supernatant_* denotes the Que content in the supernatant after digestion, and *C_digesta_* refers to the total Que content in the final digesta after simulated gastrointestinal digestion.

### 2.12. Cytotoxicity Assessment

To investigate the safety of the complex delivery system, cytotoxicity was evaluated using the CCK-8 kit. Firstly, diluted human normal colonic epithelial cells NCM460 were added to a 96-well plate and incubated in an incubator (5% CO_2_, 37 °C) for 24 h. Then, different concentrations of UOGF samples (0, 20, 50, 100, 200, 500 μg/mL) and different relative concentrations of Que (2, 5, 10 μM) of the complex samples (100 μL) were added to each well, and further incubated at 37 °C for 24 h. Finally, 20 μL of CCK-8 solution was added to wells for 2 h of incubation, and the absorbance value was read at 450 nm according to the manufacturer’s instructions and the cell viability was calculated.

### 2.13. Statistical Analysis

All experiments were conducted in triplicate, and the results were expressed as means ± SD. Statistical analysis was performed using SPSS (version 26.0) software (SPSS Inc., Chicago, IL, USA). Significant differences were determined using one-way analysis of variance (ANOVA) and Tukey’s test, with statistical significance set at *p* < 0.05.

## 3. Results and Discussion

### 3.1. Interaction Mechanism During Complex Formation

#### 3.1.1. Intrinsic Fluorescence Spectra Analysis

**Figure 1:**
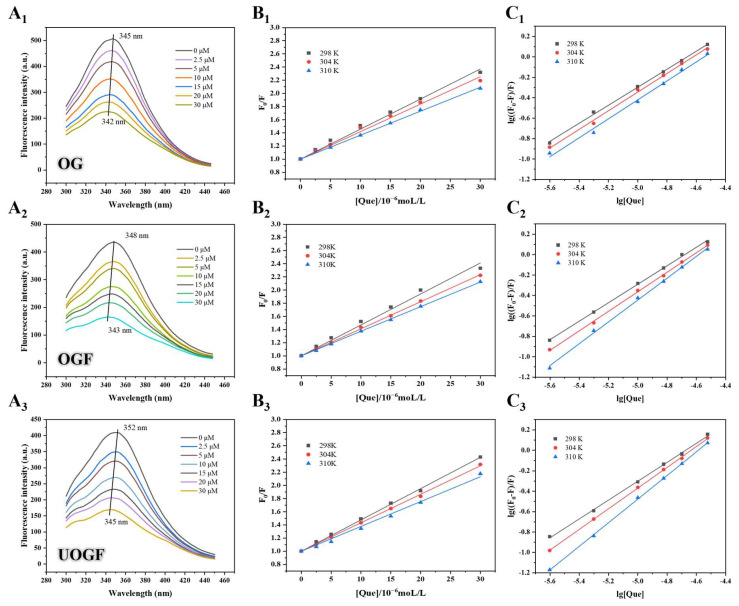
The corrected fluorescence spectra of (**A_1_**) OG, (**A_2_**) OGF and (**A_3_**) UOGF with different concentrations of Que (0–30 μM) at 298 K; Stern–Volmer plots for the quenching of (**B_1_**) OG, (**B_2_**) OGF and (**B_3_**) UOGF by Que; double-logarithmic regression plots for the quenching of (**C_1_**) OG, (**C_2_**) OGF and (**C_3_**) UOGF by Que.

To investigate the interactions between OG, OGF, and UOGF with Que, fluorescence spectroscopy was employed to characterize the microenvironmental polarity around Trp and Tyr residues in systems. As depicted in Figure 1(A_1_–A_3_), the fluorescence spectra of the OG, OGF, and UOGF complex systems at varying Que concentrations (0–30 μM) were recorded. Firstly, the differences in maximum fluorescence intensity (F_max_) and maximum emission wavelength (λ_max_) among the three systems can be ascribed to variations in the microenvironment of aromatic residues. During fibril formation, the rearrangement of peptides results in the burial of aromatic residues inside the supramolecular structure, which manifests as reduced intrinsic fluorescence [47]. With increasing Que concentration in the complex systems, a decreasing trend in Fmax was observed for all samples. This concentration-dependent fluorescence quenching indicates enhanced interaction between the proteins/fibrils and Que, which alters the molecular structure of the proteins/fibrils and restricts the exposure of intrinsic fluorophores [48]. This suggests that the binding interaction with Que induces structural alterations in OG and its two fibrillated products. Moreover, the λ_max_ values of all three complex systems exhibited varying degrees of shift with increasing Que concentration. Specifically, the λ_max_ of the OG-Que complex shifted from 345 nm to 342 nm, the OGF-Que complex shifted from 348 nm to 343 nm, and the UOGF-Que complex shifted from 352 nm to 345 nm. These blue shifts indicate that the binding interaction with Que enhances the hydrophobicity of the microenvironment surrounding the fluorescent chromophores (Trp and Tyr) in the proteins/fibrils. This further suggests that the hydrophobic regions in the protein structure may serve as the primary binding sites for Que [49].

#### 3.1.2. Fluorescence Quenching Mechanisms

To investigate the fluorescence quenching mechanism among OG, its fibrillated products, and Que, the fluorescence spectral data obtained at three distinct temperatures (298, 304, and 310 K) were fitted to the Stern–Volmer equation. The Stern–Volmer fitting curves are presented in Figure 1(B_1_–B_3_). First, the fluorescence intensity of OG, OGF, and UOGF exhibited a strong linear correlation at all tested temperatures (R^2^ > 0.99), indicating that the fluorescence quenching mechanism adheres to a single quenching process, either static or dynamic [50,51]. Generally, static quenching refers to the reduction in fluorescence intensity due to the formation of a non-fluorescent ground-state complex between the quencher and the fluorophore, whereas dynamic fluorescence quenching primarily involves excited-state reactions and collisional quenching processes [52,53]. As presented in Table 1, the fluorescence quenching parameters for the interactions between OG, OGF, UOGF, and Que were calculated. The bimolecular quenching rate constants (*K_q_*) of all three complex systems were found to be on the order of 10^12^ M^−1^s^−1^ at different temperatures, exceeding the maximum diffusion-controlled quenching constant (2 × 10^10^ M^−1^s^−1^). This suggests that the fluorescence quenching process follows a static quenching mechanism. Additionally, the type of quenching can be inferred based on temperature-dependent trends, as higher temperatures in dynamic quenching generally enhance molecular diffusion and collisional quenching [54,55]. As shown in Table 1, the Stern–Volmer quenching constants (*K_sv_*) obtained from the regression curve slopes decreased with increasing temperature, confirming a static quenching mechanism. Specifically, the *K_sv_* values of OG-Que decreased from 4.564 × 10^4^ M^−1^ to 3.642 × 10^4^ M^−1^, OGF-Que from 4.705 × 10^4^ M^−1^ to 3.740 × 10^4^ M^−1^, and UOGF-Que from 4.757 × 10^4^ M^−1^ to 3.772 × 10^4^ M^−1^. Furthermore, the *K_sv_* value of UOGF was significantly higher than that of the other samples, suggesting that Que exhibited the highest fluorescence quenching effect on UOGF, due to the higher Que binding capacity of UOGF [40]. Ultrasound indirectly affected the structural composition and self-assembly paradigm of OG fibrillation products through the unfolding of OG protein structure and the regulation of intermolecular/intramolecular interactions, which may provide an explanation for its excellent Que binding ability [25].

#### 3.1.3. Binding Mechanism of the Complex

Considering the static quenching effect between the OG protein/fibrils and the Que ligand, the binding mechanism was further analyzed using a double logarithmic Stern–Volmer equation:

(9) $$\mathrm{lg}\left(\frac{F_{0}-F}{F}\right)=\mathrm{lg}K_{a}+n\mathrm{lg}\left[Q\right]$$

where the binding constant (*K_a_*) and the number of binding sites (*n*) are key quantitative parameters reflecting the interaction between the bioactive molecule and the protein; the magnitude of *Kₐ* indicates the binding affinity between Que and the protein. A linear regression curve was plotted by fitting $\mathrm{lg}\left(\frac{F_{0}-F}{F}\right)$ against lg[*Q*], and *Kₐ* and *n* were determined from the slope and intercept of the curve, as shown in Figure 1(C_1_–C_3_).

As shown in Table 2, the binding constant (*K_a_*) of the OG-Que complex was 1.285 × 10^4^ M^−1^ at 298 K, whereas the *K_a_* values of fibrillated OGF and UOGF were significantly higher (1.714 × 10^4^ M^−1^ and 2.091 × 10^4^ M^−1^, respectively). This indicates that the fibrillation process enhanced the binding capacity for Que. Additionally, at all three temperature conditions (298, 304, and 310 K), the *K_a_* values of UOGF-Que complexes were consistently higher than those of OGF-Que, suggesting that ultrasound-assisted fibrillation further improved the binding ability with Que. Furthermore, as the temperature increased from 298 K to 310 K, the *K_a_* values of OG, OGF, and UOGF in complex with Que also increased, indicating that the formations of these three complexes were an endothermic reaction.

#### 3.1.4. Thermodynamic Parameter Analysis

To characterize the dominant interaction forces in the complex systems, the thermodynamic parameters, including entropy change (*ΔS*), enthalpy change (*ΔH*), and Gibbs free energy change (*ΔG*), were calculated using the Van’t Hoff equation:

(10) $$\ln K_{a}=-\frac{\Delta H}{RT}+\frac{\Delta S}{R}$$

(11) ΔG=ΔH−TΔS

where *K_a_* represents the binding constant between OG protein/fibrils and Que, *R* is the ideal gas constant (8.314 J/mol·K), and *T* is the absolute temperature in Kelvin (298, 304, and 310 K).

Multiple types of interactions and energy exchanges may occur among proteins, polyphenol molecules, and solvent molecules. By analyzing the thermodynamic parameters before and after the reaction, the dominant forces between the protein and ligand molecules can be determined [42,56]. As shown in Table 2, at different temperatures, the *ΔG* values for the OG, OGF, and UOGF complexes with Que were all negative, while the *ΔH* values were positive, indicating that the binding process is spontaneous and endothermic. Generally, the interaction forces between bioactive small molecules and proteins can be categorized as follows: hydrophobic interactions (*ΔH* > 0, *ΔS* > 0), electrostatic and hydrophobic interactions (*ΔH* > 0, *ΔS* < 0), hydrogen bonding and van der Waals forces (*ΔH* < 0, *ΔS* < 0), and electrostatic forces (*ΔH* < 0, *ΔS* > 0) [57,58]. In this study, both *ΔH* and *ΔS* were positive for all three complex systems, confirming that hydrophobic interactions dominated the interactions between OG protein/fibrils and Que.

#### 3.1.5. Synchronous Fluorescence Spectra

**Figure 2:**
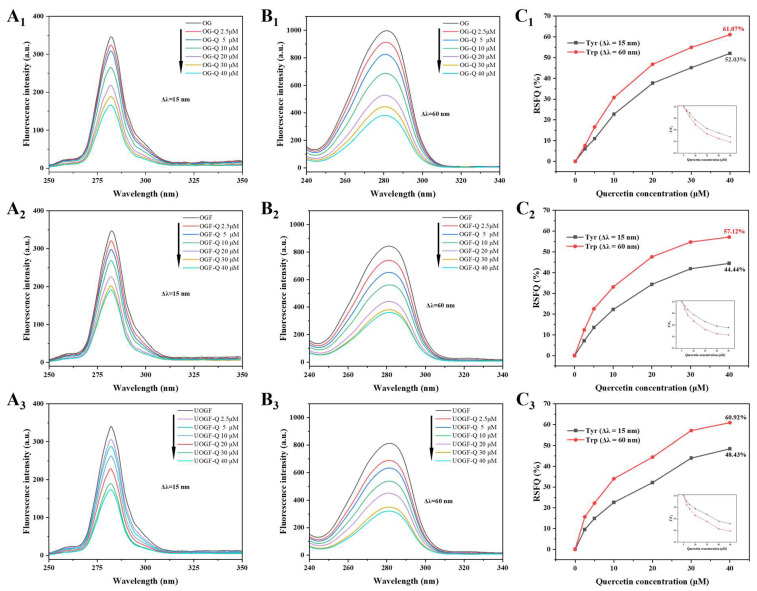
Synchronous fluorescence spectra of complex systems with increasing concentrations of Que when (**A_1_**–**A_3_**) Δλ = 15 nm and (**B_1_**–**B_3_**) Δλ = 60 nm. Comparative evaluation (**C_1_**–**C_3_**) of Que effect on the ratios of synchronous fluorescence quenching (RSFQ) of complex systems.

Synchronous fluorescence spectroscopy was employed to analyze the conformational changes in OG, OGF and UOGF. By setting wavelength intervals (Δλ) of 15 nm and 60 nm, the polarity changes in the microenvironment surrounding tyrosine (Tyr) and tryptophan (Trp) residues, respectively, were examined [59]. As shown in Figure 2(A_1_–A_3_,B_1_–B_3_), with the increasing addition of Que (0–40 μM), the fluorescence intensity corresponding to both Tyr and Trp residues consistently decreased in all sample groups, indicating that both residues are participated in the interaction between Que and OG protein/fibrils. Furthermore, the relative synchronous fluorescence quenching ratio (RSFQ = 1 − *F*/*F*_0_) was employed to assess the contributions of Tyr and Trp residues to the interaction with Que [42,60]. As illustrated in Figure 2(C_1_–C_3_), with increasing Que concentration, the RSFQ values for Trp residues in OG, OGF, and UOGF increased to 61.07%, 57.12%, and 60.92%, respectively, which were consistently higher than the corresponding RSFQ values of Tyr residues (52.03%, 44.44%, and 48.43%). These results suggest that Trp residues, being structurally closer to the interaction sites between Que and OG protein/fibrils, serve as the primary fluorescence quenching groups during the complexation process.

#### 3.1.6. Differential Fibrillation of OG Induced by Ultrasound Pretreatment

**Figure 3:**
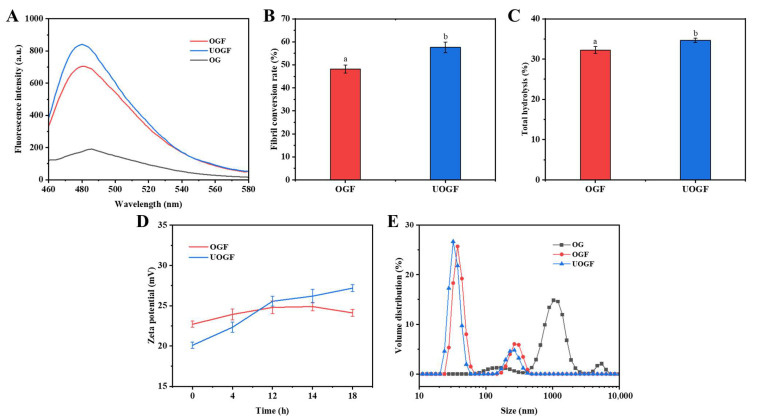
(**A**) ThT fluorescence spectra, (**B**) fibril conversion rate, (**C**) total hydrolysis after fibrillation, (**D**) zeta potential and (**E**) particle size distribution of OGF and UOGF. Letters in every panel represent statistical significance (*p* < 0.05).

Ultrasonic pretreatment may serve as a key factor underlying the differential interaction capacity of Que with protein fibril products by inducing specific structural modifications. Ultrasound facilitates structural alterations in proteins that reduce the high free-energy barriers associated with fibrillation [61]. As shown in the ThT fluorescence spectra (Figure 3A), ultrasonic pretreatment markedly increased the relative proportion of cross-β structures after fibrillation, as reflected by the maximum ThT fluorescence intensity. Consistently, the fibril conversion rate increased significantly from 48.23% in OGF to 57.61% in UOGF following ultrasonic pretreatment (*p* < 0.05) (Figure 3B), further supporting the conclusion that ultrasound promotes fibril yield. Similarly, Zong et al. (2025) confirmed that ultrasonic pretreatment assisted acid–heat incubation accelerated the hydrolysis and self-assembly of rice glutelin, as well as enhancing the final yield of rice glutelin fibrils [38]. Protein fibrillation is characterized by the denaturation and hydrolysis of proteins into peptides, followed by their self-assembly into amyloid-like fibrils [62,63]. Accelerated protein hydrolysis induced by auxiliary treatments is a major pathway facilitating fibril formation, as observed in hydrothermal, ultrasound-assisted, or microwave-assisted methods [37,64,65]. As shown in Figure 3C, the results indicated that fibril products obtained with ultrasonic pre-treatment exhibited higher degrees of hydrolysis compared with OGF produced by acid–heat treatment alone. This finding suggests that ultrasound pretreatment enhances the subsequent hydrolysis of OG during acid–thermal incubation. Consistent with previous reports, ultrasound markedly accelerated the fibrillation of β-lactoglobulin by promoting denaturation and unfolding [66]. In addition, zeta-potential measurements revealed that fibrils formed under the two different conditions displayed distinct distributions of surface-charged groups (Figure 3D), likely resulting from differences in peptide composition after hydrolysis and corresponding variations in assembly behavior. Hu et al. (2024) also demonstrated that fibrils produced by ultrasound-assisted heating exhibited unique peptide composition patterns compared with those obtained by conventional acid–heat treatment [37]. Similarly, Yang et al. (2024) confirmed that ultrasonic pretreatment not only accelerated the fibrillation kinetics of bovine serum albumin but also altered the foaming and emulsifying properties of fibrils by modulating the distribution of hydrophobic and charged groups during assembly [67]. Therefore, the higher proportion of fibrillar structures and the surface group variations derived from ultrasound-regulated assembly behavior are likely key contributors to the differential interactions with Que.

### 3.2. Delivery Performance for Quercetin

**Figure 4:**
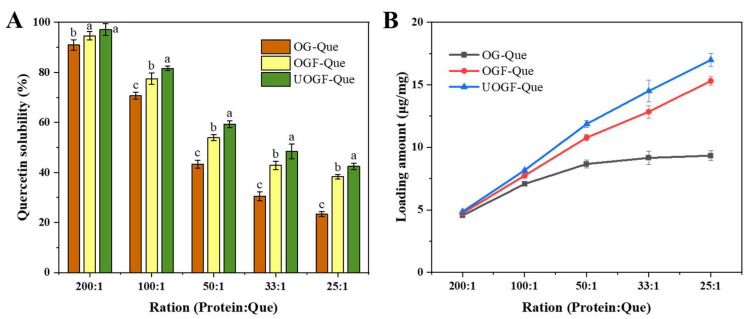
(**A**) Encapsulation capacity and (**B**) loading capacity of OG, OGF and UOGF on Que. Letters in every panel represent statistical significance (*p* < 0.05).

The entrapment efficiency and loading capacity of Que in the composite delivery system based on OG, OGF and UOGF were determined. As shown in Figure 4A, complexation with OG protein/fibrils significantly enhanced the aqueous solubility of Que. As a hydrophobic compound, Que exhibits a very low solubility in water, measured at 0.39 mg/L at 298.15 K [68]. With the protein-to-Que mass ratio of 200:1, the complexes with OG, OGF, and UOGF effectively promoted the solubility of Que. The encapsulation efficiencies of Que were 90.96%, 94.63%, and 97.16%, with corresponding loading capacities of 4.55 μg/mg, 4.73 μg/mg, and 4.86 μg/mg. However, as the mass ratio increased, the encapsulation efficiency gradually declined (*p* < 0.05). When the protein-to-Que mass ratio reached 25:1, the encapsulation efficiencies of OG, OGF, and UOGF decreased to 23.35%, 38.29%, and 42.48%, respectively. At this point, the excessive Que concentration in the system resulted in a large fraction of unencapsulated Que, which remained dispersed in the solution as microcrystals rather than being incorporated into the hydrophobic interior of the protein structure [58,69]. As depicted in Figure 4B, the loading capacity of OG, OGF, and UOGF for Que increased as the Que concentration increased. However, OGF and UOGF consistently exhibited higher loading capacities than OG, and the loading capacity growth of OG gradually plateaued. This suggests that the limited number of available binding sites in OG restricted its ability to load Que efficiently. This may be explained by the higher hydrophobicity of OG fibrils caused by ultrasound pretreatment, which facilitated the binding with Que [70]. Moreover, ultrasound pretreatment-assisted fibrillation endowed the products with differentiated surface groups, higher conversion rates, and relatively dispersed individuals, which provided more binding candidates for Que [25,40]. In conclusion, protein fibrillation significantly enhanced the binding of OG to Que in acidic media, and ultrasound-assisted fibrillation further improved the loading capacity of OG fibrils.

### 3.3. Transmission Electron Microscopy Analysis

**Figure 5:**
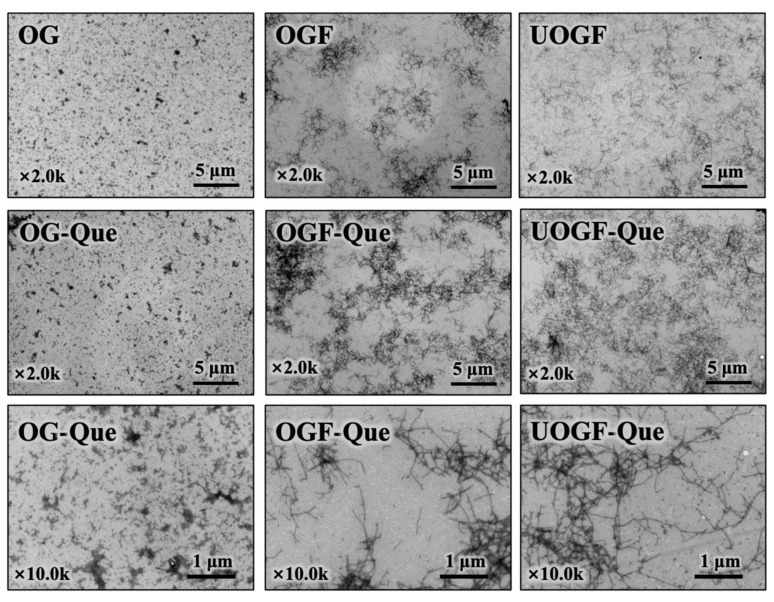
Effect of Que on microscopic morphology in complex systems.

The TEM analysis was performed to investigate the micromorphological changes in OG, OGF and UOGF during the complex formation process with Que. As shown in Figure 5, OG particles exhibited a spherical morphology, whereas OGF and UOGF displayed elongated, fibrillar structures. Upon complexation with Que, substantial morphological changes were observed. The OG-Que complex formed larger aggregated particles, whereas the fibrillar molecular network exhibited enhanced localized cross-linking and aggregation in the OGF-Que and UOGF-Que complexes. Further observations, at a higher magnification (×10k TEM images, Figure 5), revealed that the addition of Que induced the formation of dendritic protein aggregates or denser fibrillar network structures. This phenomenon can be attributed to the interaction of polyphenol molecules with reactive amino acid side chain groups, where polyphenols act as polymeric bridging agents, promoting the assembly of monomeric molecules into a structured network [71]. Similarly, previous research on the complexation of ovalbumin fibrils with resveratrol have reported that the incorporation of resveratrol promotes entanglement and aggregation, forming larger molecular clusters regardless of whether the protein existed as monomers or fibrils [27]. Another study demonstrated that EGCG effectively binds to lysozyme fibrils through hydrophobic interactions and hydrogen bonding, resulting in the formation of a densely aggregated fibrillar network [72]. Notably, no significant loss or disruption of the fibril aggregates was detected in OGF-Que and UOGF-Que images, indicating the high structural stability of the fibril molecules.

### 3.4. Hydrodynamic Diameter and Zeta-Potential Analysis

**Figure 6:**
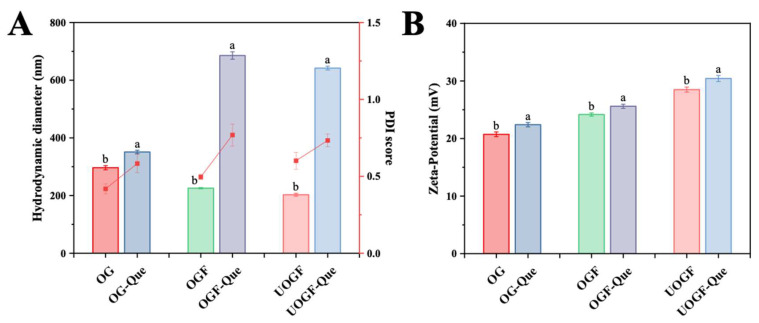
Effect of Que on (**A**) hydrodynamic diameter and (**B**) Zeta-potential in complex systems. Letters in every panel represent statistical significance (*p* < 0.05).

The hydrodynamic diameter of the OG-Que, OGF-Que, and UOGF-Que complexes were analyzed to evaluate their aggregation behavior. Although dynamic light scattering (DLS) cannot accurately quantify absolute particle size for irregularly shaped materials, it can provide valuable insights into the aggregation process. As shown in Figure 6A, the hydrodynamic diameter significantly increased after Que complexation for all samples, reaching 351.07 nm for OG-Que, 685.56 nm for OGF-Que, and 641.97 nm for UOGF-Que (*p* < 0.05). This suggests that the presence of Que facilitated the formation of larger aggregates, leading to an overall increase in solution particle size. Similar trends have been observed in previous studies, where polyphenolic compounds (such as EGCG, proanthocyanidins, apple polyphenols, and puerarin) significantly increased the particle size of WPI-based covalent complexes [73]. In another study, the complexation of rice protein with Que and resveratrol resulted in a substantial increase in particle size, whereas the complexation with curcumin did not significantly affect particle size or distribution [74]. Based on the linear structural characteristics of high aspect ratio, the available binding sites for Que on the fibril surface promoted the co-association of multiple fibril structures, leading to the emergence of agglomerates with increased hydrodynamic dimensions.

Zeta-potential is a crucial indicator of the colloidal stability of a delivery system. As shown in Figure 6B, at pH 3.2, which is below the isoelectric point (pI) of OG, the protonation of amino groups resulted in a positive surface charge (+20.73 mV for OG). The zeta-potential values for OGF and UOGF were +24.17 mV and +28.5 mV, respectively. This is consistent with previous findings, indicating fibrillation increases the exposure of charged groups, thereby enhancing the zeta-potential [30,75]. Upon complexation with Que, the zeta-potential further increased, reaching +22.40 mV for OG-Que, +25.6 mV for OGF-Que, and +30.43 mV for UOGF-Que, respectively. This observation aligns with prior studies, where polyphenol binding induced conformational modifications in proteins, exposing charged amino acid residues and increasing net positive charge [76]. Compared to OG-Que and OGF-Que, the UOGF-Que complex exhibited the most pronounced increase in zeta-potential, which may explain by its enhanced binding affinity for Que [70,77]. Therefore, the fibril-based delivery system of Que shows promoted colloidal stability due to its high zeta-potential values [13].

### 3.5. Changes in Fibril Cross-β Structures by Que

Small-molecule structural probes, such as ThT and CR, are commonly used to identify the Cross-β structures of protein fibrillation. The ThT dye binds to specific sites on the surface of amyloid fibrils, forming hydrogen bonds that enhance fluorescence [78], while CR exhibits a red shift in its ultraviolet absorption peak and birefringence under polarized light [79]. In this study, both ThT fluorescence and CR spectral shifts were employed to avoid potential interference between the exogenous compound Que and the chromophores of fibril-specific probes.

**Figure 7:**
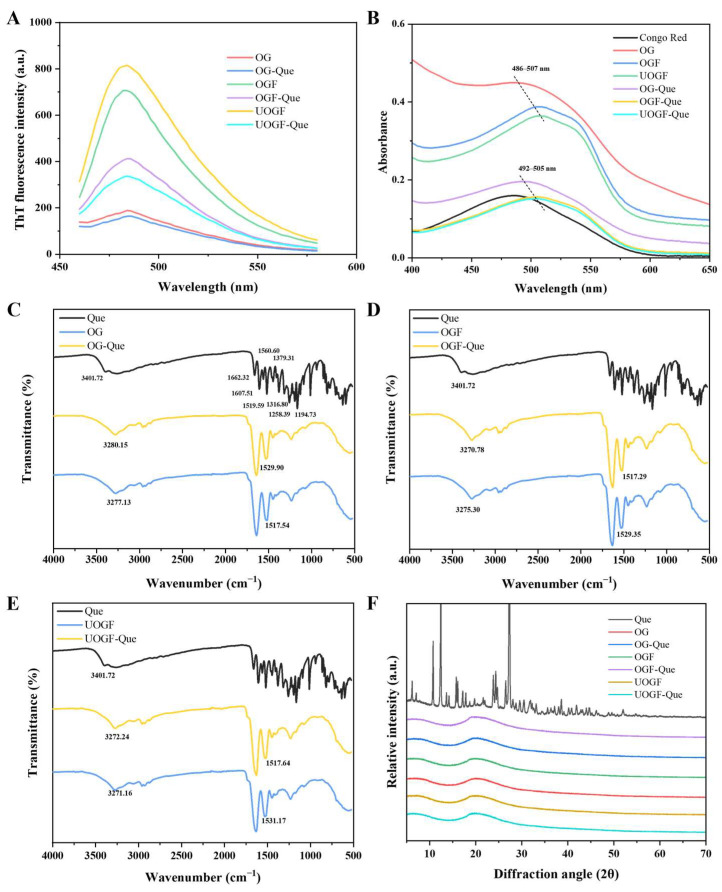
Effect of Que on structure characteristics in complex systems: (**A**) ThT fluorescence spectrum, (**B**) Congo red ultraviolet spectrum, (**C**–**E**) FTIR spectra, (**F**) XRD spectrum.

As shown in Figure 7A, the ThT fluorescence spectra of OG protein/fibrils before and after Que addition were analyzed. Compared to native OG, both OGF and UOGF exhibited significantly increased ThT fluorescence intensity after fibrillation treatment. Upon complexation with Que, the ThT fluorescence intensity of all samples decreased. This decline suggests, on the one hand, that Que induced alterations in the fibrillar structure, and on the other hand, that the formation of aggregates partially shielded the specific binding sites of the fluorescent probe, thereby reducing ThT fluorescence intensity [80]. Notably, the decrease in ThT fluorescence intensity was significantly greater in the UOGF-Que sample than in the OGF-Que sample, indicating that Que induced more pronounced structural transformations and increased protein fibril aggregation in UOGF. Similar phenomena have been previously reported: with increasing concentrations of astaxanthin and β-carotene, significant secondary structural changes in soy protein isolate fibrils were observed, accompanied by a substantial decrease in ThT fluorescence intensity, demonstrating that fibrillar structures are susceptible to the influence of exogenous substances [42,56].

Additionally, the CR ultraviolet spectra are displayed in Figure 7B. The characteristic UV absorption peak of CR in native OG was observed at 486 nm. In both OGF and UOGF samples, the absorption peak exhibited a significant red shift, confirming the formation of fibrillar structures. Upon Que addition, the UV absorbance intensity of all three systems decreased markedly. Nevertheless, the peak position still followed a sequential shift from OG-Que (formed from native OG) to OGF-Que and UOGF-Que, corresponding to the fibrillated structures. This observation indicates that the characteristic fibrillated structure was not completely disrupted, aligning with the TEM imaging results. To some extent, the attenuation of UV absorption can be attributed to the structural transformation of fibrils induced by Que, as well as fibril aggregation, which may lead to the burial of CR binding sites [79].

### 3.6. Fourier-Transform Infrared Spectroscopy Analysis

The ATR-FTIR spectroscopy was employed to gain deeper insights into the interactions between OG protein/fibril and Que, as well as to provide molecular conformational information of the complexes. As shown in Figure 7C–E, Que exhibited an FTIR spectrum consistent with previously reported data, displayed characteristic absorption peaks at 3401.72 cm^−1^ (O-H stretching vibration), 1662.32 cm^−1^ (C=O stretching vibration), 1607.51, 1560.60, and 1519.59 cm^−1^ (C=C stretching vibrations in the aromatic ring), 1379.31, 1316.80, and 1194.73 cm^−1^ (C-O stretching vibrations in C-OH groups), and 1258.39 cm^−1^ (C-O-C stretching vibration) [81,82,83,84]. In the FTIR spectra of the three complexes formed by OG, OGF, and UOGF, the characteristic benzene ring absorption peaks of Que were not retained. This suggests that Que was encapsulated within the concealed regions of the carrier structures, indicating effective embedding.

For OG, OGF, and UOGF, the absorption band at 3000–3500 cm^−1^ corresponds to O-H stretching vibrations (amide A band), while the band near 2960 cm^−1^ is predominantly associated with C-H stretching vibrations in -CH_3_ and -CH_2_ groups. The region from 1600–1700 cm^−1^ is primarily attributed to C=O stretching vibrations (amide I band), whereas the 1500–1600 cm^−1^ region corresponds to C-C and C-N stretching vibrations, as well as N-H bending in amide groups (amide II band). The 1100–1300 cm^−1^ region corresponds to the amide III band. Upon complexation with Que, the characteristic absorption peaks of OG, OGF, and UOGF in the 4000–500 cm^−1^ range exhibited varying degrees of shifts, indicating that the interaction with Que affected the molecular conformation. Evidence for hydrogen bonding between Que and C-N/N-H groups is provided by the shift in the amide A band. Specifically, the absorption peak of OG shifted from 3277.13 cm^−1^ to 3280.15 cm^−1^ in OG-Que, OGF from 3270.78 cm^−1^ to 3275.30 cm^−1^ in OGF-Que, and UOGF from 3271.16 cm^−1^ to 3272.24 cm^−1^ in UOGF-Que, confirming the presence of hydrogen bonding interactions between Que and the carrier structures. Additionally, the amide II band, which reflects hydrophobic interactions between proteins and ligands, also exhibited shifts. The absorption peak of OG shifted from 1517.54 cm^−1^ to 1529.90 cm^−1^ in OG-Que, OGF from 1529.35 cm^−1^ to 1517.29 cm^−1^ in OGF-Que, and UOGF from 1531.17 cm^−1^ to 1517.64 cm^−1^ in UOGF-Que. These shifts further confirm the presence of hydrophobic interactions between Que and the carrier structures during complex formation.

### 3.7. X-Ray Diffraction Analysis

The alterations in the crystal structure of the samples following physical or chemical treatments can be evaluated by the XRD method. As shown on Figure 7F, the XRD spectrum of free Que exhibits sharp and distinct diffraction peaks, indicating that Que possesses a characteristic crystalline structure. Moreover, the protein/fibril displays broad diffraction peaks around 10° and 20°, because the specific diffraction peaks can represent the Cross-β structure of protein fibril [25]. Additionally, no characteristic peaks corresponding to Que were detected in the spectra of the three nanocomposites (OG-Que, OGF-Que, and UOGF-Que), indicating that Que transforms from a crystalline form to an amorphous form and is successfully encapsulated. Consistent results have also been observed in other phenolic compound-based protein composite systems, such as soy protein/β-carotene composites [56], and the ovalbumin/resveratrol composites [27].

### 3.8. Functional Characteristic Evaluation

#### 3.8.1. Environmental Stability Assessment

**Figure 8:**
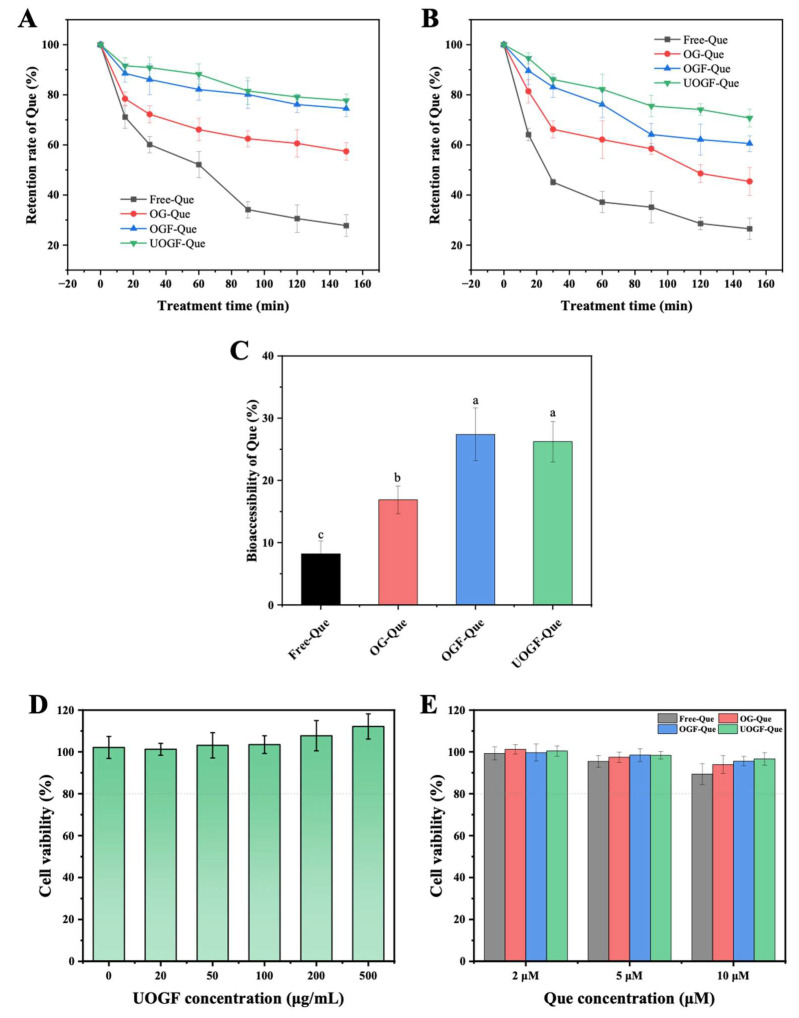
Effect of the complex systems on the (**A**) photostability, (**B**) thermal stability, (**C**) bioaccessibility of Que and (**D**,**E**) cytotoxicity evaluation. Letters in every panel represent statistical significance (*p* < 0.05).

Que exhibits photoinstability due to its unsaturated structure, which makes it prone to degradation under ultraviolet (UV) irradiation. As shown in Figure 8A, free Que underwent significant photodegradation upon UV exposure, retaining only 27.78% of its initial content after 150 min. In contrast, complex delivery systems effectively improved the photostability of Que. The retention rates of Que in OG-Que, OGF-Que, and UOGF-Que were significantly higher (*p* < 0.05), reaching 57.40%, 74.54%, and 77.23%, respectively, after 150 min of UV exposure. This enhanced photostability can be attributed to the physical barrier formed by proteins in the complexes, which effectively scattered and absorbed light, shielding Que from direct UV irradiation and preventing degradation. Furthermore, compared to OG, both OGF and UOGF provided stronger protection against Que degradation under UV irradiation. This can be explained by the fibrillation process, which exposed internal hydrophobic groups and increased surface reactive sites in the proteins, thereby enhancing their binding affinity with Que. For instance, rice glutenin fibrils exhibit stronger binding to cyanidin-3-O-glucoside (C3G) than their native form, due to the surface exposure of hydrophobic groups and positive charges, thereby improving the thermal and oxidative stability of C3G [80]. Consequently, the steric hindrance effect of OG fibrils conferred structural protection of Que, thereby preventing the oxidation of Que surface groups and blocking free radical attacks.

Thermal instability of polyphenols during food processing is another major challenge limiting the practical application of Que. As shown in Figure 8B, the retention rates of both free Que and complexed Que decreased progressively during incubation at 85 °C, indicating thermally induced degradation or oxidation. However, free Que underwent rapid degradation within the first 30 min of heating, retaining only 45.13% of its initial content. After 150 min of heating, the retention of free Que further declined to 26.48%. As expected, complexation with proteins and fibrillar forms significantly improved the thermal stability of Que. After 30 min of heating, the retention rates of Que in OG-Que, OGF-Que, and UOGF-Que were 66.21%, 83.14%, and 86.15%, respectively. After 150 min, the retention rates further increased to 45.4%, 60.54%, and 70.73%, respectively. Clearly, the protein-based complex delivery system provided effective thermal protection compared to free Que. Additionally, the fibrillated forms, OGF and UOGF, exhibited superior thermal stability due to their highly heat-resistant structures. Native globular proteins typically undergo structural denaturation upon heating, leading to disruption of their tertiary conformation and exposure of hydrophobic groups, which may weaken their interaction with Que and consequently reduce their protective effect under thermal treatment. Interestingly, UOGF exhibited better protective effects on Que than OGF. This could be attributed to the ultrasound-assisted fibrillation process, which modulated the fibrillar structure and enhanced its functional properties [25,85]. Similarly, Wang et al. found that the application of ultrasonic and enzymatic pre-treatment in the fabrication of pea protein fibrils resulted in a higher conversion rate and notable changes in fibril morphology, while also contributing to the protection of astaxanthin delivery in terms of thermal stability and bioaccessibility [86].

#### 3.8.2. Bioaccessibility Performance of Que

Bioaccessibility reflects the amount of Que present in the bile salt micellar phase following digestion [87]. Hydrophobic drugs or nutrients encapsulated within mixed micelles can be absorbed by intestinal epithelial cells [42]. As shown in Figure 8C, free Que exhibited extremely low bioaccessibility, reaching only 8.21%. This is primarily due to the poor water solubility of crystalline Que, which leads to its precipitation in the intestinal lumen and consequently limits the bioaccessibility. In contrast, the bioaccessibility of Que in OG-Que, OGF-Que, and UOGF-Que were significantly higher (*p* < 0.05), reaching 16.88%, 27.38%, and 26.23%, respectively. This enhancement in bioaccessibility is consistent with the increased solubility of Que, as complexation with proteins/fibrils significantly elevated its effective concentration in the micellar phase, thereby improving intestinal absorption. Additionally, peptides generated during protein hydrolysis may have further promoted micellization, thus enhancing solubility of Que. Moreover, OGF and UOGF were more effective in improving Que bioaccessibility, with significantly higher values than other complexes and free Que (*p* < 0.05). Given the higher encapsulation efficiency and loading capacity of UOGF-Que compared to OGF-Que, UOGF is more valuable as a delivery carrier for Que. Similarly, complexation with soy protein isolate (SPI) fibrils increased the solubility of curcumin from 95.6 μg/mL in SPI complex to 405.7 μg/mL, and significantly enhanced its bioaccessibility and digestion resistance [88]. In summary, the complexation of fibrillated OG with Que serves as an effective delivery system for hydrophobic nutrients, significantly reducing Que degradation during gastrointestinal digestion and enhancing its intestinal absorption and utilization.

#### 3.8.3. Cytotoxicity Assessment

Due to the pathological types of amyloid protein fibrils, the safety of using food protein-derived fibrils in human nutrition has also been a topic of discussion and concern in the academic community for a long time [89]. As shown in Figure 8D, no significant decrease in cell viability of NCM460 was observed in the UOGF concentration range of 0–500 μg/mL. This demonstrates that ultrasound pretreatment-assisted fibrillation modifies and regulates the structural properties of OG fibril without causing additional cytotoxic concerns [90]. Subsequently, the cytotoxicity of the three complexes (OG-Que, OGF-Que, and UOGF-Que) was investigated at the protein/fibril-to-Que mass ratio of 50:1. As shown in Figure 8E, no significant cytotoxicity was observed in the three complexes with relative concentrations of Que ranging from 2 to 10 μM, and over 90% of the cells remained viable after the treatment for 24 h. These findings enable us to preliminarily rule out the safety risks of UOGF and its complexes with Que. However, more robust evidence for the application of these complexes still requires validation through the in vivo studies.

## 4. Conclusions

Here, OG and its fibrils were utilized to construct a delivery system for hydrophobic-sensitive Que via complexation. Ultrasonic pretreatment was employed to facilitate the formation of OG fibrils and regulate the structural basis of the interaction with Que. Essentially, the complexation with Que induced dose-dependent static fluorescence quenching of the three protein/fibrils, in which hydrophobic interactions and Trp residues were the major forces for complex formation and the main fluorescence quenching groups, respectively. Among these systems, UOGF exhibited the strongest binding affinity for Que, as well as possessing the highest encapsulation and loading capacity for Que within the composite systems across various mass ratios (200:1–25:1). More importantly, the complex system formed based on UOGF exhibits significant improvement in the stability and bioaccessibility of Que. In conclusion, the integration of ultrasound pretreatment technology into the fibrillation process can reasonably improve the functional properties of protein fibrils. Furthermore, functionalizing proteins as carriers of hydrophobic active substances through fibrillation provides a new delivery strategy, facilitating the transition of protein fibrils from experimental settings to market applications. However, the in vivo fate and biological interactions of fibrils and fibril-derived products need more exploration, which is crucial for their safety in food applications.

## Figures and Tables

**Table 1:** Quenching constants for the binding of OG, OGF, and UOGF with Que at 298 K, 303 K and 310 K.

Sample	Temperature (K)	*K_sv_* (×10^4^ M^−1^)	*k_q_* (×10^−12^ M^−1^S^−1^)
OG-Que	298	4.564 ± 0.104	4.564 ± 0.104
	304	4.177 ± 0.101	4.177 ± 0.101
	310	3.642 ± 0.035	3.642 ± 0.035
OGF-Que	298	4.705 ± 0.126	4.705 ± 0.126
	304	4.113 ± 0.029	4.113 ± 0.029
	310	3.740 ± 0.024	3.740 ± 0.024
UOGF-Que	298	4.757 ± 0.050	4.757 ± 0.050
	304	4.326 ± 0.037	4.326 ± 0.037
	310	3.772 ± 0.085	3.772 ± 0.085

**Table 2:** Comparison of binding constants, binding sites and thermodynamic constants at 298 K, 303 K and 310 K.

Samples	T (K)	Binding Constants			Thermodynamic Parameters		
		*K*_a_ (M^−1^)	*n*	R^2^	*ΔG* (kJmol^−1^)	*ΔH* (kJmol^−1^)	*ΔS* (Jmol^−1^K^−1^)
OG-Que	298	1.285 × 10^4^	0.881 ± 0.016	0.9983	−23.533	15.961	132.529
	304	1.633 × 10^4^	0.912 ± 0.025	0.9964	−24.328		
	310	1.647 × 10^4^	0.928 ± 0.035	0.9930	−25.123		
OGF-Que	298	1.714 × 10^4^	0.905 ± 0.015	0.9987	−23.859	98.042	409.064
	304	2.606 × 10^4^	0.956 ± 0.014	0.9989	−26.313		
	310	7.967 × 10^4^	1.069 ± 0.023	0.9977	−28.768		
UOGF-Que	298	2.091 × 10^4^	0.924 ± 0.016	0.9985	−24.413	145.104	568.848
	304	4.987 × 10^4^	1.013 ± 0.010	0.9996	−27.826		
	310	2.026 × 10^5^	1.157 ± 0.011	0.9995	−31.239		

## Data Availability

The original contributions presented in the study are included in the article, further inquiries can be directed to the corresponding authors.
